# Bagasse Cellulose Grafted with an Amino-Terminated Hyperbranched Polymer for the Removal of Cr(VI) from Aqueous Solution

**DOI:** 10.3390/polym10080931

**Published:** 2018-08-20

**Authors:** Lu Xia, Zhonghang Huang, Lei Zhong, Fengwei Xie, Chak Yin Tang, Chi Pong Tsui

**Affiliations:** 1 School of Chemistry and Chemical Engineering, Guangxi University for Nationalities, Nanning 530006, China; leiwin@163.com (L.X.); zhfp1666@163.com (Z.H.); 2 Guangxi Key Laboratory of Chemistry and Engineering of Forest Products, Guangxi University for Nationalities, Nanning 530006, China; 3 Guangxi Key Laboratory Cultivation Base for Polysaccharide Materials and Modifications, Guangxi University for Nationalities, Nanning 530006, China; 4 Institute of Advanced Study, University of Warwick, Coventry CV4 7HS, UK; 5 International Institute for Nanocomposites Manufacturing (IINM), WMG, University of Warwick, Coventry CV4 7AL, UK; 6 Department of Industrial and Systems Engineering, The Hong Kong Polytechnic University, Hung Hom, Kowloon, Hong Kong, China; cy.tang@polyu.edu.hk (C.Y.T.); gary.c.p.tsui@polyu.edu.hk (C.P.T.)

**Keywords:** bagasse cellulose, hyperbranched polymer, adsorption capacity, Cr(VI) removal

## Abstract

A novel bio-adsorbent was fabricated via grafting an amino-terminated hyperbranched polymer (HBP-NH_2_) onto bagasse cellulose. The morphology and microstructure of the HBP-NH_2_-grafted bagasse cellulose (HBP-*g*-BC) were characterized and its adsorption capacity for Cr(VI) ions in aqueous solutions was investigated. The rough surface structure of HBP-*g*-BC that is beneficial for improving the adsorption capacity was observed by scanning electron microscopy (SEM). The grafting reaction was confirmed by Fourier-transform infrared (FT-IR) spectroscopy. The adsorbent performance was shown to be better with a lower pH value, a higher adsorbent dosage, or a higher initial Cr(VI) concentration. Moreover, the kinetics study revealed that the adsorption behavior followed a pseudo-second-order model. The isotherm results showed that the adsorption data could be well-fitted by the Langmuir, Freundlich, or Temkin models. Moreover, HBP-*g*-BC could maintain 74.4% of the initial removal rate even after five cycles of regeneration. Thus, the high potential of HBP-*g*-BC as a bio-adsorbent for heavy metal removal has been demonstrated.

## 1. Introduction

Chromium is one of the most common heavy metals used in industry and has a broad range of industrial applications, such as electroplating and metal processing. It is well-known that chromium ions Cr(VI) possess significantly higher toxicity than other forms of chromium. Without proper treatment, wastewater containing Cr(VI) may cause serious environmental problems [1,2]. Wastewater containing Cr(VI) is highly toxic to plants and animals even at low concentrations. Contact with Cr(VI) can cause ulcerations and dermatitis on the skin. Inhalation of Cr(VI) can result in serious gastrointestinal and neurological problems [3]. It has also been reported that ingestion of Cr(VI) through contaminated drinking water could potentially cause mouth or stomach cancers [4]. Therefore, the removal of Cr(VI) from wastewater is essential to protect the environment and maintain human health.

For the removal of heavy metals from wastewater, different methods, such as precipitation, solvent extraction, ion exchange, membrane filtration, and adsorption [5,6,7], have been used. The precipitation process removes heavy metals by forming precipitates that can be separated from water. This process is relatively simple and inexpensive, but sometimes causes disposal and separation problems [5]. In the solvent extraction process, organic solvents that are immiscible with water are added into wastewater to dissolve and remove heavy metals. This method is well-established and easy to operate, but it is only effective in removing certain types of metals [8]. The ion exchange process uses specific resins to exchange their cations or anions with heavy metals and remove them from wastewater. The advantages of it include high capacity, selectivity, and fast kinetics, whereas the main disadvantage is that ion exchange is not suitable for a concentrated metal solution and is highly sensitive to pH values [9]. Membrane filtration shows great potential in wastewater treatment. It can achieve a high removal efficiency and involves no phase change. The membrane filtration techniques, such as ultrafiltration (UF), reverse osmosis (RO), and nanofiltration (NF), have been used for heavy metal removals [10]. UF uses a permeable membrane of a pore size of 5–20 nm to separate heavy metals. It can work at low driving force in a small working space, but needs to overcome the adverse effects caused by membrane fouling [11]. RO utilizes a semipermeable membrane that allows for the passage of solvent and retains the metals. RO has been widely used in desalination, but its application in heavy metal removal is still limited because of the high energy consumption [5]. NF is a promising technology in membrane filtration. It provides an intermediate process between UF and RO. It is an easy, reliable, and energy-saving process with high removal efficiency. However, NF is relatively new and has some issues, such as the disposal of NF concentrate and membrane fouling, which hinder its further application [12].

The adsorption process uses adsorbents to remove heavy metals. During the adsorption, heavy metals are transferred from wastewater and adhered to the surface of the adsorbent by the physical and/or chemical interactions between them. Among various water treatment processes, adsorption is considered to be one of the most efficient techniques because of its good operational flexibility, high efficiency, and excellent reusability [13,14]. In recent years, more and more efforts have devoted to searching for low-cost and highly efficient adsorbents [15,16,17].

Cellulose is a natural biopolymer that is widely available and environmentally friendly. It has high potential to be developed into bio-adsorbents for wastewater treatment [18,19]. As cellulose contains a significant amount of hydroxyl groups, it has high affinity with heavy metal ions, which is beneficial for eliminating heavy metal ions from aqueous solutions [20,21]. Recently, the fabrication of different forms of cellulosic adsorbents and their adsorption behaviors for heavy metal ions have been widely investigated [22]. Hajeeth et al. [23] fabricated an adsorbent by grafting an acrylonitrile monomer onto cellulose extracted from a sisal fiber for the removal of Cr(VI) from aqueous solutions. The highest removal rate was 86.2%. Zhong et al. [24] utilized an adsorbent derived from wheat straw for eliminating Cu(II) and Cr(VI) from wastewater. The maximum adsorption capacity for Cu(II) and Cr(VI) was 17.1 and 62.2 mg/g, respectively. More recently, d’Halluin et al. [25] reported the use of chemically modified cellulose filter paper for the adsorption of various heavy metals in water, with a high removal rate of 90–95%.

Sugarcane bagasse is an important by-product of the sugarcane industry. There has been a growing interest in finding new applications for this cheap resource. Although the adsorption capacity of coarse sugarcane is not satisfactory, chemically modified bagasse cellulosic materials have shown great potential to be used as effective adsorbents for heavy metals [26,27,28]. Yu et al. [29] investigated the adsorption behaviors of pyromellitic-dianhydride-modified bagasse cellulose for Pb(II), Cd(II), Zn(II), and Cu(II) and found that the adsorption capacity of modified bagasse was 10 times higher than that of unmodified bagasse. Zhu et al. [30] synthesized bagasse cellulose through epoxidation and amination, which was used for the elimination of Cu(II). The adsorption capacity was 35.2 mg/g under optimal conditions. Ramos et al. [31] modified bagasse cellulose with phthalic anhydride and evaluated its adsorption performance for Co(II), Cu(II), and Ni(II). The maximum adsorption capacities obtained were 0.561, 0.935, and 0.932 mmol/g, respectively.

Hyperbranched polymers (HBPs) are a type of macromolecule with highly branched three-dimensional (3D) structures. Compared with their linear and crosslinked analogs, HBPs exhibit properties such as lower viscosity, higher solubility, and less entanglement due to their unique globular and dendritic architectures. They have been widely used in fields such as coating, nanotechnology, additives, and biomaterials [32]. Since HBPs have abundant functional groups and excellent chelation properties with various metal ions, the introduction of HBPs has recently been considered as an effective way to design and fabricate novel adsorbents for heavy metal ions, such as Cu(II), Pb(II), Zn(II), and Cr(VI) [33,34]. Zang et al. [35] reported an adsorbent prepared by modifying cotton fibers with HBPs, which showed removal rates for Cu(II) and Pb(II) to be about 73.5 and 71.2 wt %, respectively. Lin et al. [36] investigated the performance of a hyperbranched polyamide-modified corncob adsorbent for Cr(VI), and the adsorption capacity was shown to be up to 47.8 mg/g. More recently, Li et al. [37] modified chitosan with an amino-terminated hyperbranched polymer, which was applied to remove Cr(VI). The removal rate was shown to be up to 93.8%. Despite these previous efforts, there is still a variety of natural biopolymers which have strong potential to be used for heavy metal adsorbents and remain to be tested. Biopolymers from agricultural residues, such as sugarcane bagasse, rice straws, corncobs, and wheat straws, have the prominent advantages of being used as biosorbent materials owing to their low cost, good processibility, and high capacity. In particular, sugarcane bagasse is heavily produced and has a high proportion of cellulose [38]. Grafting with an amino-terminated HBP will be an effective way to improve the adsorption performance of sugarcane bagasse cellulose. However, there are limited studies focusing on sugarcane bagasse cellulose modified with HBPs and its adsorption behavior and mechanism as an adsorbent for heavy metal removal.

This work aimed at fabricating a novel bio-adsorbent based on sugarcane bagasse cellulose (BC) grafted with an amino-terminated hyperbranched polymer (HBP-NH_2_) and evaluating the adsorption capacity of this bio-adsorbent for Cr(VI) ions in aqueous solutions. The morphology and microstructure of the fabricated HBP-NH_2_-grafted bagasse cellulose (HBP-*g*-BC) were characterized by SEM and FT-IR. The effects of pH, adsorbent dosage, and initial Cr(VI) concentration on the adsorption behavior of the bio-adsorbent were also examined. Moreover, the adsorption mechanism was explored by a kinetics and an isotherm analysis.

## 2. Materials and Methods

### 2.1. Materials and Chemicals

Coarse sugarcane bagasse was supplied by Nanning Sugar Industry Co., Ltd. (Nanning, China). Anhydrous sodium sulfate (AR), sodium periodate (AR), and ethylene glycol (AR) were purchased from Kelong Chemical Reagent Factory (Chengdu, China). Diethylenetriamine (AR), methyl acrylate (AR), methanol (AR), glacial acetic acid (AR), and anhydrous ethanol (AR) were purchased from Sinopharm Chemical Reagent Co., Ltd. (Shanghai, China). Potassium dichromate (AR) was supplied by Guanghua Chemical Reagent Factory (Shantou, China). Deionized water was made in the lab and used in all the experiments.

### 2.2. Preparation of Bagasse-Based Adsorbent

#### 2.2.1. Synthesis of Dialdehyde Bagasse Cellulose (DABC)

Dialdehyde bagasse cellulose (DABC) was prepared according to Huang et al. [39]. Coarse bagasse cellulose was firstly ground and purified by reaction with anhydrous sodium sulfate. Then, the purified BC was mixed with sodium periodate. Based on our preliminary experiments, the synthesis conditions used were the temperature of 35 °C, the reaction time of 3.5 h, the pH of 3, and the mass ratio of sodium periodate to cellulose of 2:1, which could allow abundant aldehyde groups to be generated. Then, 5 mL of ethylene glycol was added into the mixture of cellulose and periodate for another 1 h of reaction. Afterwards, the mixture was filtered and washed with deionized water and anhydrous ethanol three or four times to obtain the solids. Finally, DABC powder was obtained by vacuum-drying the solid at 40 °C until reaching a constant weight.

#### 2.2.2. Synthesis of HBP-NH_2_

HBP-NH_2_ was prepared by the polycondensation procedure introduced by Wang et al. [40]. An amount of 52 mL of diethylenetriamine was added to a three-necked flask and cooled by submerging in an ice cooling bath. Then, under the protection of nitrogen, 43 mL of methyl acrylate and 100 mL of methanol were mixed and slowly poured into a flask to react at room temperature. After 3 h of reaction, AB_2_ and AB_3_ monomers were obtained. These two types of monomers were transferred to an eggplant flask attached to a rotary evaporator to react. During the reaction, firstly the methanol was removed by decompression. Then, the temperature was increased to 150 °C and the reaction was carried on under decompression for another 4 h to obtain HBP-NH_2_.

#### 2.2.3. Synthesis of HBP-NH_2_-Grafted Bagasse Cellulose (HBP-*g*-BC)

Ten grams (10 g) of dried DABC powders and 100 mL of methanol were added to a three-necked flask. They were mixed by stirring and heating at 45 °C. When the temperature of the solution reached 45 °C, 5 mL of glacial acetic acid was added for another 20–30 min of reaction. Then, 40 g of an HBP-NH_2_ methanol solution (the volume ratio of HBP-NH_2_ to methanol was 1:1) was added to react with DABC for 7 h to obtain HBP-*g*-BC. Afterwards, the obtained cellulose was washed with deionized water and ethanol and was then filtered by suction to form a filter cake. The final product was obtained by vacuum-drying the filter cake under 40 °C until it reached a constant weight.

### 2.3. Fourier-Transform Infrared (FT-IR) Spectroscopy

The Fourier-transform infrared (FT-IR) spectra of BC, DABC, and HBP-*g*-BC were measured by a Fourier-transform infrared spectrometer (Nicolette Magna 550II, GMI, Ramsey, MN, USA). A small amount of sample was mixed with KBr powder. Then, the blend was ground and compressed to form the testing disc. The spectral range of 4000–400 cm^−1^ was used for analysis of each sample.

### 2.4. Microscopy

The surface morphologies of BC and HBP-*g*-BC were observed by a field emission scanning electron microscope (Zeiss, Supra 55, Oberkochen, Germany) with an accelerating voltage ranging from 0.02 to 30 kV and a resolution of 0.8 nm.

### 2.5. Thermogravimetric Analysis

The thermal stabilities of BC and HBP-*g*-BC were measured by a simultaneous thermal analyzer (DSC/DTA-TG, STA 449 F3 Jupiter, Netzsch, Selb, Germany). The samples were heated from 35 to 900 °C with a heating rate of 10 °C/min.

### 2.6. Adsorption Study

#### 2.6.1. Adsorption Procedure

Solutions were prepared to simulate wastewater produced from a metal processing unit [41]. A certain amount of potassium dichromate was dissolved in deionized water. The pH value of Cr(VI) solution was adjusted by addition of HCl or NaOH.

To conduct a batch mode adsorption test, a certain amount of HBP-*g*-BC was added to the prepared Cr(VI) solution at a certain temperature and a pH value. The adsorption was carried out in a rotary shaker at a speed of 200 rpm/min for different time periods.

A series of batch adsorption tests were conducted to investigate the effects of pH, adsorbent dosage and initial Cr(VI) concentration on the adsorption performance of HBP-*g*-BC. For each set of conditions, the measurements were carried out in triplicate for different samples to ensure the reproducibility of the data. The mean values were calculated and used for further analysis.

#### 2.6.2. Determination of Adsorption Capacity

The metal ion adsorption capacity of HBP-*g*-BC was determined by the adsorption amount *q* and the removal rate *R*, which can be calculated by the following two equations.

(1) $$q=\frac{(C_{0}-C_{e})V}{M}\;$$

(2) $$R=\frac{\left(C_{0}-C_{e}\right)}{C_{0}}\times 100\%\;$$

In these equations, *C*_0_ and *C_e_* are the concentrations (mg/L) of metal ions in the solution before and after the adsorption, respectively, *V* is the volume (L) of the aqueous solution, and *M* is the mass (g) of HBP-*g*-BC consumed during the adsorption.

### 2.7. Regeneration Study

For adsorption, 0.1 g of HBP-*g*-BC was added to 50 mL of Cr(VI) solution (20 mg/L) at 35 °C and pH = 3 in a conical flask. The adsorption process was carried out for 4 h. Afterwards, the adsorbent was treated with 0.1 mol/L HCl for 24 h and was then filtered. The filtrate was used for desorption analysis. Then, the adsorbent was washed with distilled water to remove the residual acid and to ready it for the next desorption test. The above procedure was repeated for five cycles to evaluate the reusability of the adsorbent.

## 3. Results and Discussions

### 3.1. FT-IR Analysis of Unmodified and Modified Bagasse Celluloses

**Figure 1:**
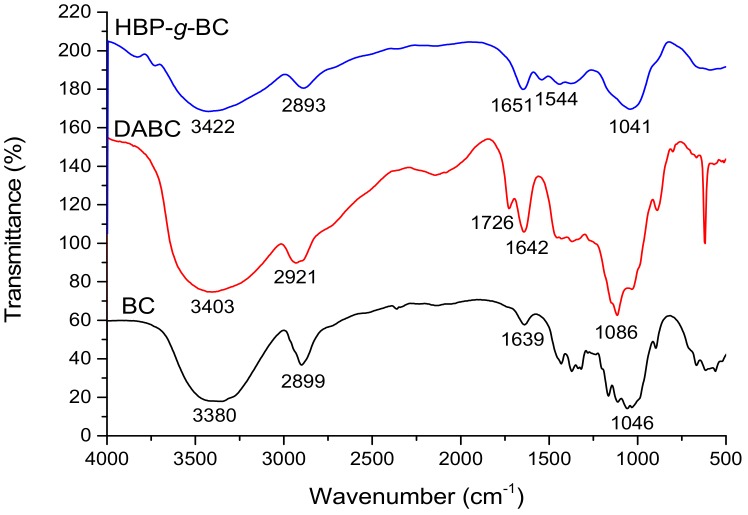
FT-IR spectra of Bagasse Cellulose (BC), Dialdehyde Bagasse Cellulose (DABC), and HBP-NH_2_-Grafted Bagasse Cellulose (HBP-*g*-BC).

The FT-IR spectra of BC, DABC, and HBP-*g*-BC are shown in Figure 1. It can be seen that the shapes of these three curves are similar. The broad band observed around 3400 cm^−1^ is considered to be due to the hydroxyl (–OH) stretching. The characteristics peak around 2900 cm^−1^ could be ascribed to the C–H vibration. Compared with that of BC, the spectrum of DABC shows a characteristic adsorption peak of carbonyl group at 1726 cm^−1^, suggesting that aldehyde groups were introduced to BC to form DABC [39]. In the spectrum of HBP-g-BC, this peak of aldehyde group disappears, the peak of C–H vibration at 2921 moves to 2893 cm^−1^, and there is a new characteristic peak at 1544 cm^−1^, which could be assigned to the vibration of amine groups. This indicates that through the reaction between the aldehyde groups of DABC and the amino groups of HBP-NH_2_, HBP-NH_2_ was successfully grafted onto BC to form HBP-*g*-BC [37].

### 3.2. SEM of Unmodified and Modified Bagasse Celluloses

**Figure 2:**
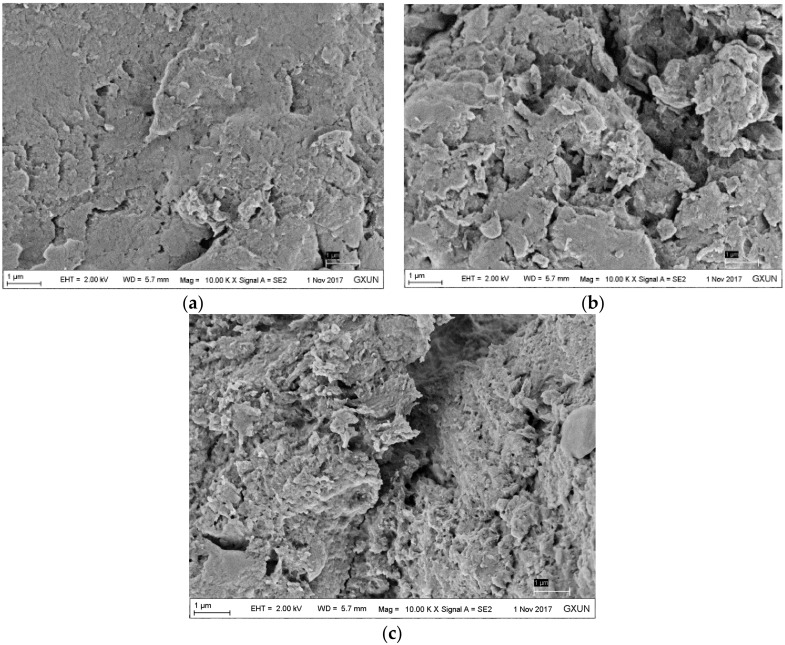
SEM images of BC (**a**), DABC (**b**), and HBP-*g*-BC (**c**).

The SEM images of BC, DABC, and HBP-*g*-BC are presented in Figure 2. At the same magnification, while the surface of BC (without modification) was smooth and compact, the modification made the surface become loose and rough. Furthermore, the surface of HBP-*g*-BC had some small peaks with irregular shapes. This surface feature presenting a higher specific surface area could lead to an increased number of active adsorption sites and thus an improved adsorption capacity of HBP-*g*-BC.

### 3.3. TGA of BC and HBP-g-BC

**Figure 3:**
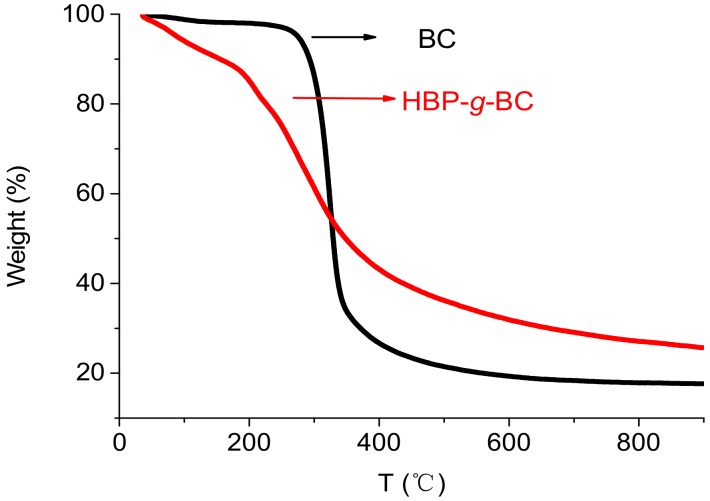
TGA curves of BC and HBP-*g*-BC.

Figure 3 shows the TGA results of BC and HBP-*g*-BC. Three stages of weight loss can be observed for both BC and HBP-*g*-BC. At the initial stage (35–242 °C for BC and 35–172 °C for HBP-*g*-BC), the weight loss of BC and HBP-*g*-BC was 2.7% and 11.2%, respectively. This first-stage weight loss could be mostly ascribed to the moisture evaporation. The major weight loss occurred at the second stage (242–400 °C for BC and 172–400 °C for HBP-*g*-BC), during which the weight losses for BC and HBP-*g*-BC reached 73.6% and 56.9%, respectively. This second-stage weight loss could be attributed to the degradation and decomposition of BC and HBP-*g*-BC. The onset temperature for the significant weight loss of HBP-*g*-BC was lower than that for BC. Regarding this, the introduction of HBP-NH_2_ groups might make the polymer easier to decompose. The results are consistent with those in previous studies, where the decomposition temperature of cellulose usually decreased by grafting with hyperbranched polymers [37].

## 4. Evaluation of Adsorption Capacity of HBP-*g*-BC

### 4.1. Effect of Solution pH

**Figure 4:**
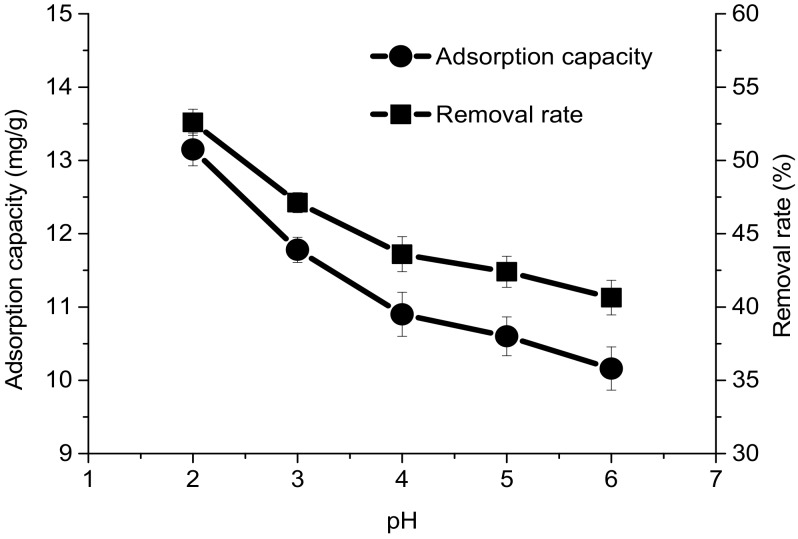
Effect of solution pH on the adsorption capacity and removal rate for Cr(VI).

The pH of an aqueous solution significantly affects the ionic forms of heavy metals and the surface charge of an adsorbent in the solution. Therefore, pH strongly influences the adsorption capacity of the adsorbent for Cr(VI) ions. Cr(VI) is more reactive at low pH values because its reduction potential decreases with increasing pH. In our preliminary experiments, the change in adsorption capacity is small when the pH value is higher than 6. To investigate the effect of pH, the adsorption tests were carried out at pH values from 2 to 6 [37]. The relationship between the adsorption capacity for Cr(II) and the pH is shown in Figure 4. It can be seen that with the increased pH, both the adsorption capacity and the removal rate decreased. As previously reported about a chitosan-based bio-adsorbent, a higher Cr(VI) adsorption capacity usually occurs at a pH value lower than 4 [37]. The greater adsorption capacity for Cr(VI) at a low pH value might be explained by the surface protonation of HBP-*g*-BC. The protonation of amino groups was more active under acidic conditions. In this case, the surface of HBP-*g*-BC was strongly positively charged. Moreover, since Cr(VI) mainly exists in the solution in the forms of HCrO_4_^−^ and CrO_4_^2−^ at low pH values, the strong electrostatic attraction makes it easy for the adsorbent to capture negatively charged Cr(VI) ions, leading to a higher adsorption capacity. On the contrary, high pH values usually reduce the degree of protonation of amino groups, which might negatively influence the adsorption performance of HBP-*g*-BC.

### 4.2. Effect of Adsorbent Dosage

**Figure 5:**
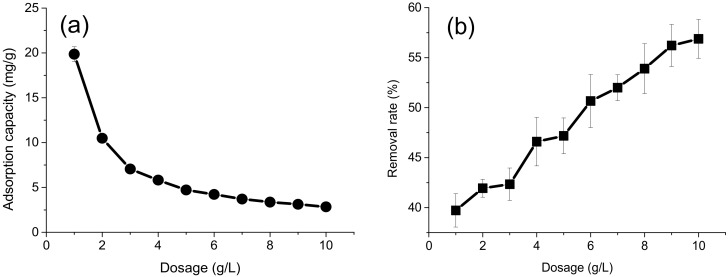
Effect of adsorbent dosage on the adsorption capacity (**a**) and removal rate (**b**) for Cr(VI).

Adsorbent dosage is an important parameter that should be evaluated and controlled in the wastewater treatment. The results (Figure 5) show that the adsorption capacity for Cr(VI) decreased with increasing the dosage of the adsorbent from 1 to 10 g/L, which might be due to the unsaturation of active sites on the surface of each adsorbent [30,37]. However, the removal rate of Cr(VI) increased with increasing the dosage of the adsorbent. This is as expected since, with a higher amount of the adsorbent, the total surface area is increased and thus there are more active sites available for the adsorption [23].

### 4.3. Effect of Initial Cr(VI) Concentration

**Figure 6:**
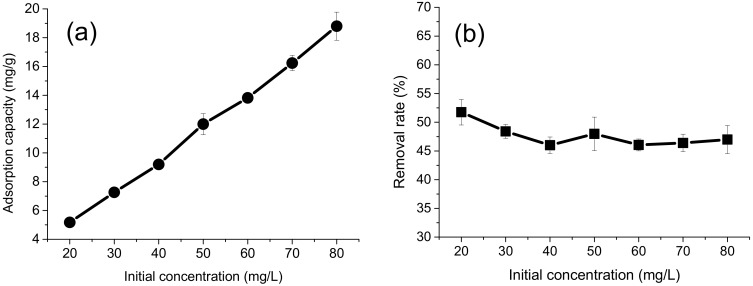
Effect of initial Cr(VI) concentration on the adsorption capacity (**a**) and removal rate (**b**) for Cr(VI).

Figure 6 shows the adsorption capacity and removal rate as a function of the initial concentration of Cr(VI) between 20 and 80 mg/L. It was found that the adsorption capacity increased with an increase in the initial Cr(VI) concentration. When the initial concentration was 80 mg/L, the adsorption capacity reached 18.80 mg/g, 3.6 times higher than the capacity when the initial concentration was 20 mg/L, which was 5.18 mg/g. Regarding this, when the initial concentration is higher, there is a larger concentration gradient and the driving force for transferring the metal ions is also stronger. This provides more opportunities for Cr(VI) ions to be in contact with the adsorbent. Accordingly, more ions will be attached to the active sites on the adsorbent surface, leading to a higher adsorption capacity. The removal rates for different initial Cr(VI) concentrations were 51.75%, 48.40%, 46.00%, 47.98%, 46.05%, 46.39%, and 46.99%, respectively. This suggests that HBP-*g*-BC is an excellent adsorbent that can maintain a stable removal rate by increasing its adsorption capacity with increasing initial concentration.

### 4.4. Adsorption Kinetics

The adsorption kinetics measurements were conducted at 25, 35, and 45 °C. To investigate the kinetics of the adsorption process, the pseudo-first-order model (Equation (3)) and the pseudo-second-order model (Equation (4)) were used to evaluate the experimental data [42]. The equations of these models are given as follows:

(3) $$\ln(q_{e}-q_{t})=\ln q_{e}-k_{1}t\;$$

(4) $$\frac{t}{q_{t}}=\frac{1}{k_{2}q_{e}^{2}}+\frac{t}{q_{e}}\;$$

In these equations, *q_e_* and *q_t_* (mg·g^−1^) are the amount of the Cr(VI) ions adsorbed at equilibrium and time *t* (min), respectively; *k*_1_ (min^−1^) is the pseudo-first-order rate constant of the adsorption; and *k*_2_ (mg·min^−1^) is the pseudo-second-order rate constant of the adsorption.

**Figure 7:**
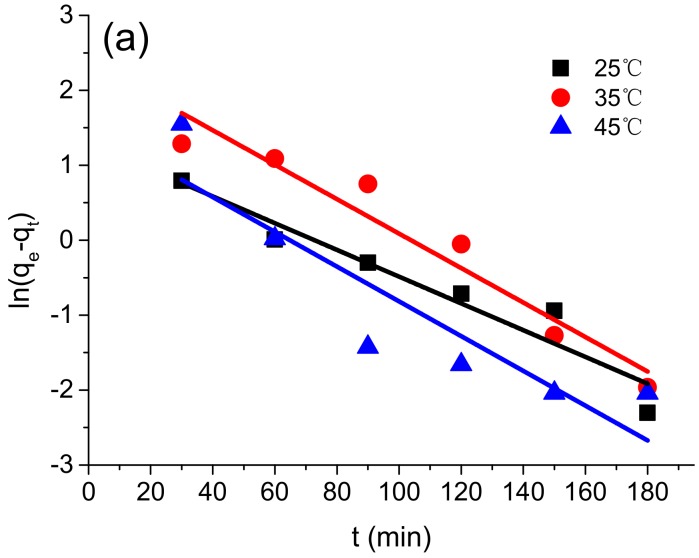

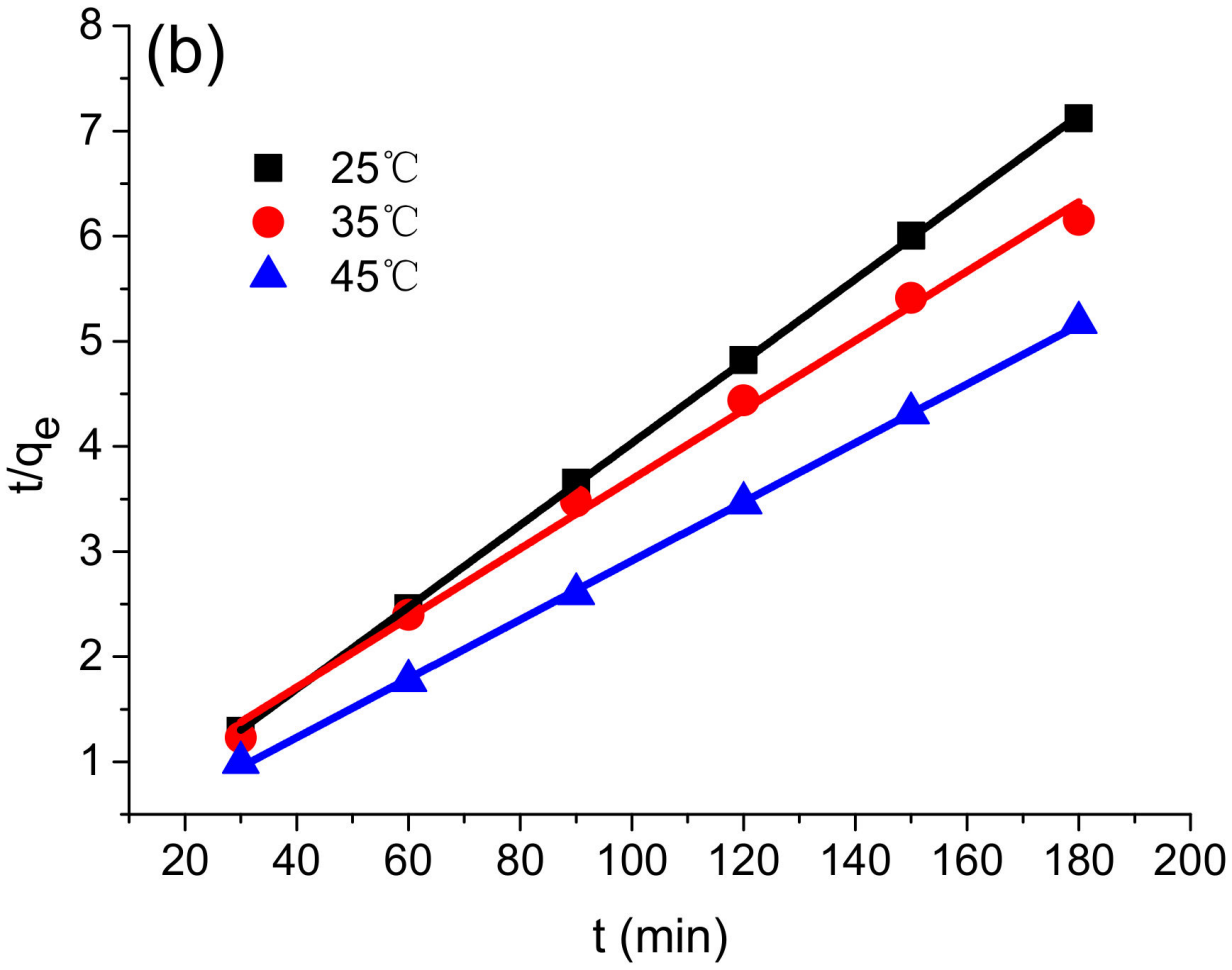
Fitting with the pseudo-first-order model (**a**) and the pseudo-second-order model (**b**).

As shown in Figure 7a, the pseudo-first-order model fails to describe the kinetics of the adsorption process for Cr(VI). The correlation coefficients for 25, 35, and 45 °C were 0.9062, 0.9216, and 0.7793, respectively. A previous study also showed that for chemically modified cellulose used for Ag(I), Pb(II), Cd(II), Ni(II), Zn(II), Sn(II), and Cu(II) adsorptions, the pseudo-first-order model was not suitable [25]. However, as shown in Figure 7b, the adsorption process can be accurately fitted with the pseudo-second-order model, with the correlation coefficients of 0.9999, 0.9941, and 0.9998, respectively, for 25, 35, and 45 °C. This suggests that the Cr(VI) adsorption process might mainly be a chemical adsorption process, which involves valence forces generated by sharing or exchanging electrons between metal ions and the bio-adsorbent [43].

### 4.5. Adsorption Isotherms

The adsorption isotherm model provides a relationship between the amount of adsorbed metal ions and that of the non-adsorbed metal ions in a solution when the equilibrium was reached. It is critical for predicting the adsorption behavior and comparing the adsorbent performance under different conditions [44]. To thoroughly investigate the adsorption isotherm of the HBP-*g*-BC adsorbent, three widely used isotherm models, namely Langmuir (Equation (5)), Freundlich (Equation (6)), and Temkin (Equation (7)), were used to examine the experimental data [44]:

(5) $$\frac{1}{q_{e}}=\frac{1}{q_{\max}}+\left(\frac{1}{q_{\max}K_{L}}\right)\frac{1}{C_{e}}\;$$

(6) $$\ln q_{e}=\ln K_{F}+\frac{1}{n}\ln C_{e}\;$$

(7) $$q_{e}=\left(\frac{RT}{b_{T}}\right)\ln(A_{T})+\left(\frac{RT}{b_{T}}\right)\ln(C_{e})\;$$

In these equations, *q_e_* (mg/g) is the amount of metal ions adsorbed at the equilibrium; *C_e_* (mg/L) is the concentration of metal ions in the solution at the equilibrium; *K_L_* is the Langmuir constant (L/mg); *K_F_* and 1/*n* are the Freundlich constants related to the capacity and intensity of the adsorption, respectively; *A_T_* (L/g) is the Temkin equilibrium constant; *b_T_* is the Temkin constant associated with the adsorption heat; *R* (8.314 J/mol) is the gas constant; and *T* (K) is the absolute temperature.

**Figure 8:**
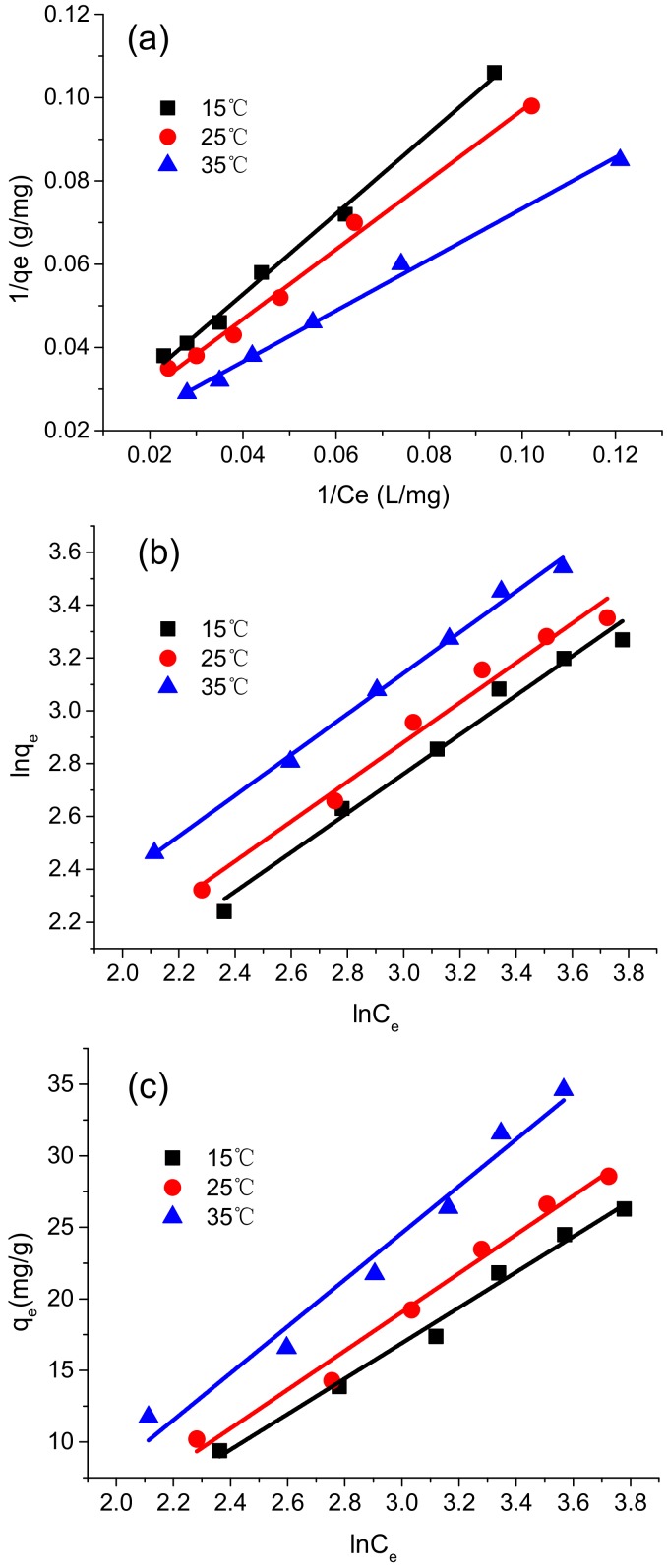
Fitting with isotherm models at different temperatures: Langmuir model (**a**); Freundlich model (**b**); and Temkin model (**c**).

The isotherm model parameters for Cr(VI) ions under three different temperatures are summarized in Table 1 and the regression curves for these three models are plotted in Figure 8. It can be seen that all the three models can properly fit the adsorption process, with high correlation coefficients achieved. The well-fitting of the Freundlich model indicates that the physical adsorption existed during the adsorption process. This might be due to the rough and irregular surface structure of HBP-*g*-BC, which could easily lead to multilayer physical adsorption on its surface [24]. The Langmuir model also gave a good fit to the measurements. This means that monolayer chemical adsorption of Cr(VI) ions occurs on the outer surfaces of the adsorbent, which was energetically homogeneous [30]. The Temkin model could accurately describe the adsorption process as well, meaning that there were strong interactions between the adsorbed metals and the adsorbent during the adsorption [25].

The constant *R*_L_ expressed as *R*_L_ = 1/(1 + *K*_L_*C*_0_) was calculated using the Langmuir model. *R*_L_ can be used to effectively evaluate whether the adsorption isotherm process is favorable (0 < *R*_L_ < 1) or unfavorable (*R*_L_ > 1). The *R*_L_ values for 15, 25, and 35 °C were 0.505, 0.472, and 0.417, respectively, indicating that the adsorption process was favorable. This result corresponds to the *n* values (1.34, 1.33, and 1.30) calculated by the Freundlich model, since *n* values in the range of 1 to 10 also suggest a favorable adsorption process. Furthermore, with the increased temperature, both *q*_max_ and *K*_L_ in the Langmuir model were increased. This suggests that the adsorption of Cr(VI) by HBP-*g*-BC was an endothermic process, which means a higher temperature can effectively accelerate the adsorption process.

These results are in agreement with previous research [37]. The possible adsorption process of Cr(VI) by HBP-*g*-BC might occur in three consecutive stages. Firstly, anionic Cr(VI) ions bind with the positively charged amino groups of HBP-*g*-BC. Secondly, Cr(VI) reduces to Cr(III) by adjacent electron-donor groups. Finally, Cr(III) reacts with the functional groups on HBP-*g*-BC to form a Cr(III) complex. It is worth noting that the maximum adsorption capacity of HBP-*g*-BC for Cr(VI) at 25 °C was 75.36 mg/g. In our preliminary studies, the maximum adsorption capacity of DABC was 11.27 mg/g under the same conditions. It is evident that the grafting of HBP-NH_2_ significantly improved the adsorption capacity of BC.

### 4.6. Desorption and Regeneration

**Figure 9:**
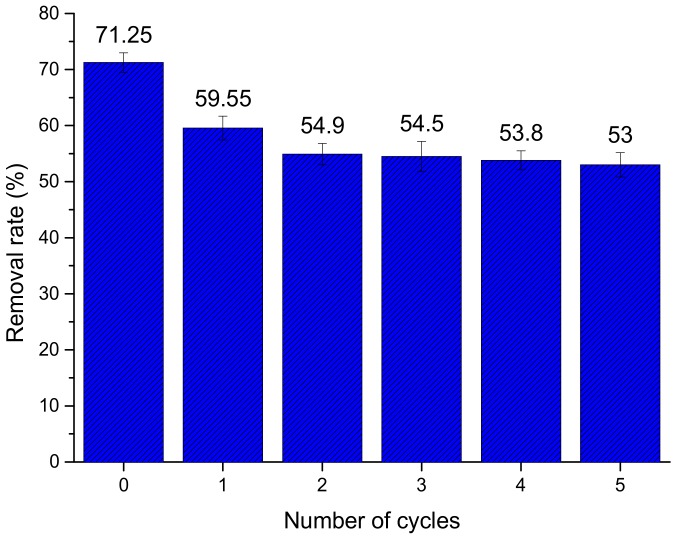
Removal rates of HBP-*g*-BC after different cycles of regeneration.

The removal rates for Cr(VI) of regenerated HBP-*g*-BC after up to five consecutive adsorption–desorption cycles are presented in Figure 9. It is found that the initial removal rate was 71.25%. After the first recovery cycle, the removal rate decreased to 59.55%. The reason might be that some of the functional groups were destroyed by HCl during the first recovery process. After the second cycle, the removal rate became stable. After five cycles, the removal rate was 53%, which was 74.4% of the initial removal rate (71.25%). This suggests that HBP-*g*-BC had good reusability and thus has great potential to be used as an economical adsorbent.

## 5. Conclusions

Here, we have demonstrated that the HBP-*g*-BC bio-adsorbent is capable of removing Cr(VI) ions from aqueous solutions, and its maximum adsorption capacity for Cr(VI) was much higher than that of DABC. The introduction of HBP-NH_2_ groups onto BC could lead to a rough and irregular surface, which is instrumental to Cr(VI) adsorption. We also found that the adsorption performance was highly dependent on the pH, adsorbent dosage, and initial concentration of Cr(VI). A high adsorption capacity could be achieved at a low pH value due to the surface protonation of HBP-*g*-BC. The removal rate was increased with a higher adsorbent dosage as a higher amount of the adsorbent could provide more active adsorption sites. With the increased initial Cr(VI) concentration, the adsorption capacity was also increased. Regarding this, the stronger driving force at larger concentration gradients could facilitate the contact between Cr(VI) ions and the adsorbent. We found that the adsorption kinetics data can be accurately described by the pseudo-second-order model rather than by the pseudo-first-order model. The adsorption isotherm data are in good agreement with the Freundlich, Langmuir, and Temkin models, which means that the adsorption of Cr(VI) is an endothermic and favorable process. Moreover, HBP-*g*-BC could maintain 74.4% of the initial removal rate even after five cycles of regeneration. Thus, our work has shown the high potential of HBP-*g*-BC as a bio-adsorbent for heavy metal removal and wastewater treatment.

## Figures and Tables

**Table 1:** Isotherm model parameters of the Langmuir, Freundlich, and Temkin models for HBP-*g*-BC.

Isotherm Model	Parameters	15 °C	25 °C	35 °C
Langmuir	*q*_max_ (mg/g)	71.43	75.36	82.99
	*K*_L_ (L/mg)	0.014	0.016	0.020
	*R*_L_	0.505	0.472	0.417
	R^2^	0.9949	0.9916	0.9941
Freundlich	*K*_F_	1.703	1.876	2.286
	*n*	1.34	1.33	1.30
	R^2^	0.9784	0.9778	0.9946
Temkin	RT/*b*_T_	12.413	13.556	16.348
	*A*_T_	0.194	0.203	0.224
	R^2^	0.9876	0.9811	0.9694
